# Avian Influenza A (H5N1) Age Distribution in Humans

**DOI:** 10.3201/eid1303.060849

**Published:** 2007-03

**Authors:** Matthew Smallman-Raynor, Andrew D. Cliff

**Affiliations:** * University of Nottingham, Nottingham, England; † University of Cambridge, Cambridge, England

**Keywords:** Avian influenza A H5N1, age distribution, humans, letter

**To the Editor:** A total of 229 confirmed human cases of avian influenza A (H5N1) were reported to the World Health Organization (WHO) from 10 countries of Africa, Asia, and Europe in the 30 months leading up to July 4, 2006 (*1*). WHO has highlighted the skewed age distribution of these confirmed cases toward children and young adults, with relatively few cases in older age categories (*2*). An explanation for this age bias is currently lacking, although a range of behavioral, biological, demographic, and data-related factors may account for the observed pattern (*2*,*3*).

To determine whether the statistical parameters of the case distribution can shed any light on the issue, we reviewed the age profile of patients with confirmed avian influenza A (H5N1) included in WHO’s Situation Updates—Avian Influenza archive (January 13, 2004–May 18, 2006) (*4*). We supplemented our review with case information from an additional WHO source (*5*); to allow for the age structure of reporting countries, we accessed age-specific population estimates for 2005 from the Population Division of the United Nations Secretariat (*6*).

For the period under review, age-related information was available for 169 case-patients with WHO-confirmed human avian influenza A (H5N1) in 10 countries. Information for an additional 47 confirmed case-patients, reported to WHO from Vietnam (n = 39) and Turkey (n = 8), could not be ascertained from the published sources. The mean age of the 169 sample case-patients (77 males and 92 females) was 19.8 years (median 18.0; range 0.3–75.0). Age distribution was as follows: 0–9 years, 26.0%; 10–19 years, 29.0%; 20–29 years, 23.1%; 30–39 years, 16.0%; and ≥40 years, 5.9%. Estimated age-specific case rates per million population were 0.15 (0–9 years), 0.15 (10–19 years), 0.13 (20–29 years), 0.08 (30–39 years), and 0.02 (≥40 years).

Box-and-whisker plots (*7*) (Figure) illustrate the skewed nature of the age distribution of cases by sex (A), year of report (B), and patient outcome (C); the third quartiles of the distributions (Q_3_, defined by the box tops) demarcate an age band (30–35 years) above which proportionally few cases (<10%) occurred. The country-level analysis in plot D yields similar findings, although interpretation is limited by the small numbers of cases (<10) in some countries (Azerbaijan, Cambodia, Djibouti, Iraq, and Turkey). Examination of case-patients in the 30- to 39-year age category showed a pronounced “front-loading” effect, with 21 case-patients 30–35 years of age and only 6 case-patients 36–39 years of age.

**Figure:**
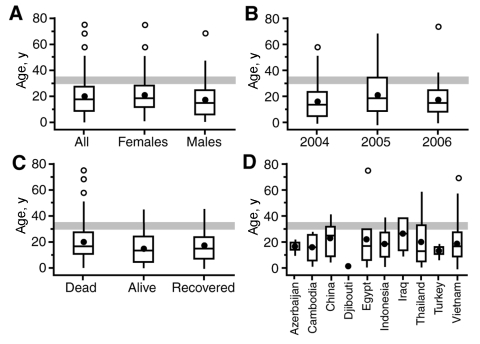
Age distribution of patients with confirmed cases of avian influenza (H5N1), December 2003–May 2006 (*4*,*5*). Box-and-whisker plots show the age distribution of patients by A) sex; B) year of report, C) patient outcome, and D) country. The horizontal line and bullet mark in each box give the median and mean age of cases, respectively. Variability in age is shown by plotting the first and third quartiles (Q_1_ and Q_3_) of the ages as the outer limits of the shaded box. Whiskers encompass all ages that satisfy the criteria Q_1_ – 1.5(Q_3_ – Q_1_) (lower limit) and Q_3_ + 1.5(Q_3_ – Q_1_) (upper limit). Points beyond the whiskers denote outliers. Panel C data are based on the recorded status of patients according to World Health Organization sources, with the category “alive” formed to include patients who were last reported as hospitalized or discharged. The age band 30–35 years is marked on each graph for reference.

Subject to multiple selection biases in the identification and reporting of WHO-confirmed human cases of avian influenza A (H5N1) (*2*), our analysis yields 3 noteworthy observations: 1) case counts and case rates suggest similar levels of disease activity in the age categories 0–9, 10–19, and 20–29 years; 2) few cases have occurred above the age band of 30–35 years; and 3) the skewed distribution of cases toward children and young adults transcends sex, reporting period, patient outcome, geographic location, and, by implication, local cultural and demographic determinants.

Behavioral factors increase the risk for exposure in younger persons and have been proposed as 1 determinant of the age distribution of confirmed human cases of avian influenza (H5N1) (*2*). However, the possible role of biologic (immunologic and genetic) and other factors has yet to be determined (*3*). Such factors may include an age-related bias in case recognition, in which clinical suspicion about the cause of respiratory disease in older persons is lower. Alternatively, we suggest that the 3 observations listed above are consistent with a biological model of geographically widespread immunity to avian influenza A (H5N1) in persons born before 1969, i.e., ≈35 years before the onset of the currently recognized panzootic in domestic poultry. Such a model would account for the similar rates of disease activity in younger age categories, the sudden and pronounced reduction of cases in patients >30–35 years of age, and the age skew that transcends the sociocultural and demographic contexts of countries and continents.

The results of broad serologic surveys for antibodies to influenza A (H5N1) virus, suggestive of a cohort effect or otherwise, have yet to be published, although anecdotal reports of completed surveys point to a lack of widespread human infection with the virus (*8*). Current evidence indicates that pandemic influenza of humans since 1918 has been restricted to 3 influenza A virus subtypes: H1 (1918–57 and 1977–present); H2 (1957–68); and H3 (1968–present) (*9*,*10*). If an element of immunity to avian influenza A (H5N1) does exist in older populations, its possible association with geographically widespread (intercontinental) influenza A events before the late 1960s merits further investigation.

## References

[1] World Health Organization. Cumulative number of confirmed human cases of avian influenza A (H5N1) reported to WHO: 4 July 2006. Geneva: The Organization; 2006. Available from http://www.who.int/csr/disease/avian_influenza/country/en/index.html

[2] World Health Organization. Epidemiology of WHO-confirmed human cases of avian influenza A (H5N1) infection. Wkly Epidemiol Rec. 2006;81:249–57. 16812929

[3] World Health Organization. Avian influenza fact sheet (April 2006). Wkly Epidemiol Rec. 2006;81:129–36. 16673509

[4] World Health Organization. Situation updates–avian influenza. Geneva: The Organization; 2004–6. Available from http://www.who.int/csr/disease/avian_influenza

[5] World Health Organization. Avian influenza: assessing the pandemic threat. Geneva: The Organization; 2005.

[6] Population Division of the Department of Economic and Social Affairs of the United Nations Secretariat. World population prospects: the 2004 revision; and world urbanization prospects: the 2003 revision. New York: United Nations; 2006. Available from http://esa.un.org/unpp

[7] Tukey JW. Exploratory data analysis. Reading (MA): Addison-Wesley; 1977.

[8] Enserink M. Avian influenza: amid mayhem in Turkey, experts see new chances for research. Science. 2006;311:314–5. 10.1126/science.311.5759.314 16424301

[9] Dowdle WR. Influenza A virus recycling revisited. Bull World Health Organ. 1999;77:820–8. 10593030 PMC2557748

[10] Hilleman MR. Realities and enigmas of human viral influenza: pathogenesis, epidemiology and control. Vaccine. 2002;20:3068–87. 10.1016/S0264-410X(02)00254-2 12163258

